# Reducing non-attendance in outpatient appointments: predictive model development, validation, and clinical assessment

**DOI:** 10.1186/s12913-022-07865-y

**Published:** 2022-04-06

**Authors:** Damià Valero-Bover, Pedro González, Gerard Carot-Sans, Isaac Cano, Pilar Saura, Pilar Otermin, Celia Garcia, Maria Gálvez, Francisco Lupiáñez-Villanueva, Jordi Piera-Jiménez

**Affiliations:** 1 Catalan Health Service, Barcelona, Spain; 2 Digitalization for the Sustainability of the Healthcare System DS3 – IDIBELL, Barcelona, Spain; 3 Faculty of Informatics, Telecommunications and Multimedia, Universitat Oberta de Catalunya, Barcelona, Spain; 4 Universitat Politècnica de Catalunya, Barcelona, Spain; 5 Hospital Clinic de Barcelona, Institut d’Investigacions Biomèdiques August Pi i Sunyer (IDIBAPS), Barcelona, Spain; 6 Department of Medicine, Universitat de Barcelona (UB), Barcelona, Spain; 7 Faculty of Medicine, Universidad Alfonso X El Sabio, Madrid, Spain; 8 Badalona Serveis Assistencials, Badalona, Spain; 9 Department of Information and Communication Sciences, Universitat Oberta de Catalunya, Barcelona, Spain

**Keywords:** Non-attendance, No show, Clinical decision making, Delivery of healthcare, Access to healthcare, Decision trees

## Abstract

**Background:**

Non-attendance to scheduled hospital outpatient appointments may compromise healthcare resource planning, which ultimately reduces the quality of healthcare provision by delaying assessments and increasing waiting lists. We developed a model for predicting non-attendance and assessed the effectiveness of an intervention for reducing non-attendance based on the model.

**Methods:**

The study was conducted in three stages: (1) model development, (2) prospective validation of the model with new data, and (3) a clinical assessment with a pilot study that included the model as a stratification tool to select the patients in the intervention. Candidate models were built using retrospective data from appointments scheduled between January 1, 2015, and November 30, 2018, in the dermatology and pneumology outpatient services of the Hospital Municipal de Badalona (Spain). The predictive capacity of the selected model was then validated prospectively with appointments scheduled between January 7 and February 8, 2019. The effectiveness of selective phone call reminders to patients at high risk of non-attendance according to the model was assessed on all consecutive patients with at least one appointment scheduled between February 25 and April 19, 2019. We finally conducted a pilot study in which all patients identified by the model as high risk of non-attendance were randomly assigned to either a control (no intervention) or intervention group, the last receiving phone call reminders one week before the appointment.

**Results:**

Decision trees were selected for model development. Models were trained and selected using 33,329 appointments in the dermatology service and 21,050 in the pneumology service. Specificity, sensitivity, and accuracy for the prediction of non-attendance were 79.90%, 67.09%, and 73.49% for dermatology, and 71.38%, 57.84%, and 64.61% for pneumology outpatient services. The prospective validation showed a specificity of 78.34% (95%CI 71.07, 84.51) and balanced accuracy of 70.45% for dermatology; and 69.83% (95%CI 60.61, 78.00) for pneumology, respectively. The effectiveness of the intervention was assessed on 1,311 individuals identified as high risk of non-attendance according to the selected model. Overall, the intervention resulted in a significant reduction in the non-attendance rate to both the dermatology and pneumology services, with a decrease of 50.61% (*p*<0.001) and 39.33% (*p*=0.048), respectively.

**Conclusions:**

The risk of non-attendance can be adequately estimated using patient information stored in medical records. The patient stratification according to the non-attendance risk allows prioritizing interventions, such as phone call reminders, to effectively reduce non-attendance rates.

**Supplementary Information:**

The online version contains supplementary material available at 10.1186/s12913-022-07865-y.

## Background

Non-attendance, defined as a missed appointment without prior notification, is an important obstacle for adequate management of healthcare centers. High non-attendance rates are associated with increased waiting lists and healthcare and societal costs, as well as reduced effectiveness and efficiency of the healthcare system [1, 2]. At the patient level, missed appointments may lead to inadequate follow-up and late diagnosis or complication management, thus increasing the health risk of non-attendees. Reported non-attendance rates worldwide are highly heterogeneous and range from 13.2% (average countries in Oceania) to 43.0% (Africa); the estimated average rate in Europe is 19.3% [3].

Various authors have proposed interventions to reduce the harmful effects of non-attendance, such as overbooking [4] and open access [5], or to improve attendance rates directly, for example, by providing information, reminders, and incentives to patients [6–8]. Of them, the use of appointment reminders based on short message services (SMS) and telephone calls have been widely used [9–11]. Although current evidence suggests equal effectiveness of both interventions, reported results are heterogeneous, and most studies have low-quality design [10].

Regardless of the reminding strategy, identifying patients at higher risk of non-attendance may reduce costs and resources, thus increasing the sustainability of the intervention. The determinants of non-attendance are complex and may include patient-related factors (e.g., age and gender), their previous attendance history, and factors associated with the given appointment (e.g., lapse from schedule date, and weekday and season of the appointment) [12, 13]. In the last few years, a growing number of models for predicting no-shows have been proposed; however, most of them achieved an accuracy lower than the attendance rate [14]. The poor performance may be attributed to multiple factors that challenge model development, such as the type of data available or the sample size. Furthermore, the high variability of non-attendance rates worldwide suggests that behavioral determinants of non-attendance and the effectiveness of mitigating measures may depend on the country and healthcare system organization. Unlike traditional statistics for predicting outcomes, which rely on predetermined equation as a model, machine learning algorithms adaptively improve their performance as the number of samples available for learning increases. These techniques are particularly suitable for predicting complex outcomes, such as those that depend on human behavior [15, 16].

Therefore, we aimed to develop a machine learning model for predicting patients’ non-attendance and assess the effectiveness of selective phone calls to patients at high risk of non-attendance according to the resulting model.

## Methods

### Overview of study design

This study was conducted at two outpatient services (i.e., dermatology and pneumology) of the *Hospital Municipal de Badalona* (Spain) and included three stages: (1) the development of a non-attendance predictive model for each outpatient service, (2) the prospective validation of the resulting models, and (3) a pilot study to assess the effectiveness of integrating the predictive model into the organization of the healthcare provider.

Candidate models were developed using retrospective data from appointments scheduled between January 1, 2015, and November 30, 2018. Data were randomly assigned to one of the following two sets: 75% of the collected data were used for model building and algorithm training, and the remaining 25% were used in a retrospective validation of the model. The predictive capacity of the selected model was then validated prospectively using data from appointments scheduled between January 7 and February 8, 2019. Finally, we conducted a pilot study to assess the effectiveness of a preventive intervention based on selective phone call reminders to patients identified as high-risk of non-attendance according to the selected model. The pilot study was conducted between February 25 and April 19, 2019.

All data, including retrospective information for model building and prospective information of the pilot study, were collected in a pseudonymized way and handled according to the General Data Protection Regulation 2016/679 on data protection and privacy for all individuals within the European Union and the local regulatory framework regarding data protection. The pilot study included in this report was not intended to change biomedical or health-related outcomes; therefore, the research committee of Badalona Serveis Assistencials waived the need for ethics committee approval.

### Variables collected for model development and validation

We collected three types of variables from the Electronic Medical Record database: sociodemographic characteristics of patients, characteristics of the appointment, and history of patients’ attendance. Sociodemographic characteristics included gender, age, nationality, marital status, and home address, which was used to calculate the distance from the patient’s home to the hospital. Characteristics of the appointment included hour, weekday, month, type of visit (first, second, successive), the reason for the visit, treatment category, physician, lead time (days since scheduling until the appointment date), and rescheduling. Variables regarding the record of patient’s attendance included the history of previous attendance, number of prior visits, days since the last appointment, and the last appointment status.

### Predictive model development and validation

We conducted bivariate analyses to identify relationships between the available variables and non-attendance and correlations between covariates to rule out strong interactions. All variables with a significant association with non-attendance were included in training algorithms based on the following models: decision trees, XGBoost, Support Vector Machines (SVM), and k-nearest neighbor (kNN). For each learning algorithm, a 5-fold cross-validation and a grid search for hyperparameter optimization was used in the training, considering all significant variables. Class imbalance (approximately, 80% of attendees and 20% of non-attendees) was addressed by stratified random sampling.

The performance of the obtained model was retrospectively assessed using the dataset reserved to this end. Because the model was intended to identify patients at high risk of non-attendance, specificity, defined as the proportion of real non-attendees among all identified by the algorithm as high-risk , was used for measuring performance. Sensitivity (i.e., the proportion of real attendees among low-risk patients) and accuracy (i.e., the proportion of appointments predicted correctly) were also estimated. Model selection was based on a balance between (1) maximizing specificity and accuracy and (2) the explanability and interpretability of the algorithms. The model performance in predicting non-attendance was prospectively validated using the same definitions of performance as for the retrospective validation. The only exception was considering balanced accuracy instead of raw accuracy because of class imbalance in the prospective validation.

### Pilot study

The pilot study included all consecutive patients with at least one appointment scheduled between February 25 and April 19, 2019, in either of the two involved services. The primary endpoint of the pilot study was the reduction of the non-attendance rate among patients at high risk of non-attendance according to the predictive model obtained. The week before the appointment, patients who were considered at high risk of non-attendance were randomly assigned to either a control or intervention group, balanced regarding age and gender. Right after randomization (i.e., one week before the appointment), patients allocated in the intervention group received a reminder phone call (up to three contact attempts) in which they were encouraged to either attend or early cancel the visit, whereas those in the control group did not receive any reminder. The outcomes related to the appointment reminder (i.e., whether the patient was reached, appointment cancellation or rescheduling, appointment attendance) were recorded. A post-intervention self-guided debriefing session was conducted on April 26, 2019, following a 3-phase conversational structure, including reaction, analysis and summary phases [17]. Two dermatology and two pneumology specialists, together with the responsible of administrative management and three directors (Medical Officer, Information Officer and Management Officer) participated in the conversation.

### Statistical analysis

Continuous variables were presented as the mean and standard deviation (SD), and categorical variables as frequency and percentage. Non-attendance rates were calculated by dividing the number of non-attended visits by the number of scheduled visits on a given period. Data from remote appointments, and negative days of waiting time (i.e., introduced in the program after the visit) were excluded from the analysis. Specificity, sensitivity, and accuracywere estimated directly from the contingency table of predicted and real missed appointments, whereas balanced accuracy was calculated as (sensitivity+specificity)/2. For variable selection, categorical variables were compared using the Chi-Square test, whereas continuous variables were compared using analysis of variance (ANOVA). Correlations between quantitative variables were analyzed using the Pearson correlation test, whereas correlations between qualitative variables were analyzed with Cramer’s V coefficient. The significance threshold was set at a bilateral alpha value of 0.05. All analyses were performed using the R software (version 3.6.1).

## Results

### Variable analysis

Non-attendance algorithms were developed using data from 33,329 appointments scheduled in the dermatology service and 21,050 in the pneumology service. The global non-attendance rates of these appointments were 20.90% and 18.37% for dermatology and pneumology outpatient services, respectively. When comparing the sociodemographic characteristics, appointment characteristics and attendance history of patients who attended the appointment in the dermatology outpatient service and those who not, significant differences were observed in all variables except gender and marital status (Table S2, Supplementary file 1). Similarly, all variables showed a significant association with non-attendance in appointments in the pneumology outpatient service, except gender, physician, and number of reschedules (Table S3). We found no strong correlations between variables, neither categorical nor numerical (Table S4).

### Model and prediction performance

**Fig. 1:**
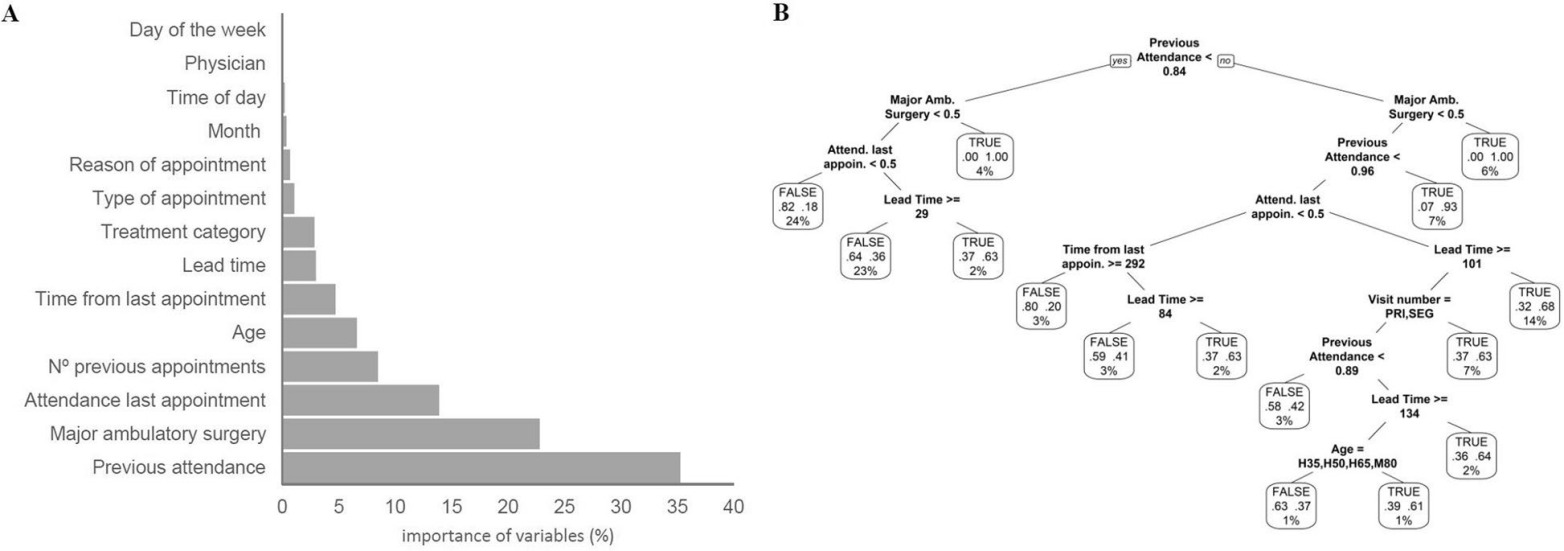
Dermatology model for predicting the non-attendance risk. **A** Relative importance of variables, according to the Gini index. **B** Decision tree representation; each leaf includes the following information: probability of the model (true: > 0.5; false: < 0.5), probability of each class within the node (values between 0 and 1), and percentage of observations of the node

**Fig. 2:**
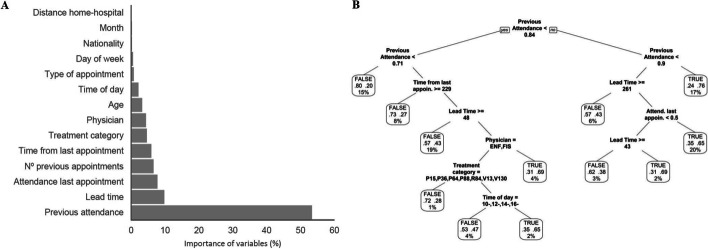
Pneumology model for predicting the non-attendance risk. **A** Relative importance of variables, according to the Gini index. **B** Decision tree representation; each leaf includes the following information: probability of the model (true: > 0.5; false: < 0.5), probability of each class within the node (values between 0 and 1), and percentage of observations of the node

After assessing both (1) the specificity, sensitivity, and accuracy,and (2) the explanability and interpretability of four training algorithms, we selected the decision trees algorithm for model development. The algorithm kNN yielded unacceptable results in terms of sensitivity, whereas XGBoost and SVM resulted in similar metric performance values to those of decision trees. Table S1 (Supplementary file 1) summarizes the performance values of each model. Figures 1A and 2A show the design of the resulting predictive models for dermatology and pneumology outpatient services, respectively. In the dermatology predictive model, the patient’s history of previous attendance was the most relevant factor to predict non-attendance in the future, followed by major ambulatory surgery, the status of the last appointment, number of prior visits, and age (Fig. 1B). This model displayed a specificity of 79.90%, a sensitivity of 67.09%, and an accuracy of 73.49%. Similarly, in the pneumology predictive model, the patient’s previous attendance history was also the most important variable to predict non-attendance, followed by lead time, the status of the last appointment, number of prior visits, and number of days since the last visit Fig. 2B. The specificity, sensitivity, and accuracy of this model were 71.38%, 57.84%, and 64.61%, respectively.

### Model validation

The prospective validation of the non-attendance predictive models included 758 and 637 appointments in the services of dermatology and pneumology, respectively. In the dermatology service, the predictive model identified 348 (45.91%) appointments at high risk (i.e., ≥50% likelihood) of non-attendance, 123 of which were actually missed appointments. The total number of real non-attendances was 157, thus yielding a specificity of the model of 78.34% (95%CI 71.07, 84.51). The sensitivity and balanced accuracy of this model were 62.56% (95%CI 71.07, 84.51) and 70.45%, respectively. Correspondingly, 283 (44.43%) appointments scheduled in the pneumology service were identified as high risk of non-attendance, 81 of which were missed appointments. The total amount of real non-attendances was 116, resulting in a specificity of 69.83% (95%CI 60.61, 78.00). The sensitivity and balanced accuracy of the pneumology model were 61.23% (95%CI 56.89, 65.43) and 65.53%, respectively. Compared with the retrospective validation used during model development, specificity in the prospective validation was reduced by approximately 1.5 percentage points.

### Pilot study

During the study period, 1,311 individuals had at least one appointment to either the dermatology or pneumology outpatient services that was identified as high risk non-attendance according to the selected model. Among them, 1,108 (805 and 303 in the dermatology and pneumology services, respectively) had available data and were, therefore, included in the analysis. Of the 805 patients with scheduled visits in the dermatology service, 390 (48.45%) were allocated to the intervention group and 415 (51.55%) to the control group. Correspondingly, 303 individuals had scheduled visits to the pneumology service, 146 (48.18%) and 157 (51.82%) allocated in the intervention and control groups, respectively. Table 1 summarizes the baseline characteristics of the individuals enrolled in the pilot study. None of the variables showed significant differences between control and intervention groups, except the time from the last visit among individuals visited at the pneumology service, which was higher in the intervention group than in the control group.

**Table 1:** Baseline characteristics of the pilot study population

	***Dermatology*** *(N=805)*			***Pneumology*** *(N=303)*		
	Control	Intervention	*P*	Control	Intervention	*P*
**Sociodemographic characteristics**						
Age group (years), *n (%)*						
0-14	24 (5.78)	28 (7.18)	0.971	1 (0.64)	0 (0.00)	0.071
14-18	24 (5.78)	24 (6.15)		2 (1.27)	0 (0.00)	
18-25	31 (7.47)	31 (7.95)		6 (3.82)	0 (0.00)	
25-35	47 (11.33)	36 (9.23)		9 (5.73)	3 (2.05)	
35-50	93 (22.41)	89 (22.82)		20 (12.74)	24 (16.44)	
50-65	74 (17.83)	67 (17.18)		54 (34.39)	47 (32.19)	
65-80	81 (19.52)	79 (20.26)		54 (34.39)	62 (42.47)	
> 80	41 (9.88)	36 (9.23)		11 (7.01)	10 (6.85)	
Gender (male), *n (%)*	177 (42.65)	169 (43.33)	0.901	92 (58.60)	94 (64.38)	0.360
**Attendance history**						
Attended to the last visit, *n (%)*	281 (70.96)	284 (75.94)	0.340	120 (76.92)	116 (80.56)	0.355
Major ambulatory surgery*, mean (SD)*	0.16 (0.36)	0.16 (0.37)	0.773	-	-	-
Rate of attendance of previous appointments, *mean (SD)*	0.75 (0.19)	0.76 (0.17)	0.853	0.77 (0.11)	0.78 (0.10)	0.499
Lead time (days)^a^, *mean (SD)*	125.35 (102.1)	123.68 (98.47)	0.814	188.03 (165.64)	216.45 (170.64)	0.142
N° of previous visits, *mean (SD)*	42.96 (45.76)	44.37 (48.78)	0.681	69.93 (62.27)	70.33 (60.89)	0.955
Time from last visit (days), mean (SD)	141.53 (312.56)	169.11 (407.8)	0.291	67.32 (73.37)	128.95 (338.93)	0.028

^a^Days of waiting since scheduling until the appointment date

In the dermatology setting, 267 (68.46%) individuals allocated in the intervention arm were successfully contacted by phone. From which, 251 attended the appointment, and 16 missed it (non-attendance rate 5.99%). Regarding the pneumology service, 95 (65.07%) individuals of the intervention group were successfully contacted; 86 of them attended the appointment, and 9 did not (non-attendance rate 9.47%). Table 2 summarizes the non-attendance rate of each group in each clinical setting. Overall, the interventions applied resulted in a significant decrease of the non-attendance rate for both dermatology and pneumology services, with a reduction of non-attendance of 50.61% and 39.33%, respectively. In both services, non-attendance rates were significantly lower among individuals in the intervention group that were successfully contacted than those who could not be reached (79.54% and 62.85% reductions for dermatology and pneumology services, respectively).

**Table 2:** No-shows in the pilot study, *No. (%)*

	Dermatology (*N*=805)	*P*	Pneumology (*N*=303)	*P*
**Control group**	112 (26.99)	< 0.001	39 (24.84)	0.048
**Intervention group**^a^	52 (13.33)		22 (15.07)	
Not reached	36 (29.27)	<0.001	13 (25.49)	0.019
Contacted	16 (5.99)		9 (9.47)	

^a^Received a reminder telephone call one week before the date of the appointment

All participant of the post-study debriefing consistently perceived the intervention as successful. However, two issues were identified: (1) the overload of the hospital agenda after preventing non-shows, and (2) the overburden of the administrative staff associated with phone calls to patients at high risk of non-attendance.

## Discussion

We found that the models that better predicted non-attendance in dermatology and pneumology outpatient services were based on decision trees and included the following variables: patient’s history of previous attendance, major ambulatory surgery, status of the last appointment, number of previous visits, and age, for dermatology, and patient’s history of previous attendance, lead time, status of the last appointment, number of previous visits, and number of days since the last visit, for pneumology. The use of the prediction models to identify individuals at high risk of non-attendance for further selective phone call reminders allowed reducing in approximately 50% and 40% the non-attendance rate in dermatology and pneumology services, respectively.

The systematic review conducted by Carreras et al. showed that at least half of the studies on no-show prediction identified age, gender, distance from home to the healthcare center, weekday, visit time, lead time, and history of previous attendance as predictors of non-attendance; marital status and visit type (first or successive) were also frequently used [14]. Our findings were mostly in line with the results reported by Carreras et al., although we did not find an association between gender and non-attendance, as reported elsewhere [18, 19]. Other studies described that non-attendance was associated with the number of previous appointments [20, 21], the status of the last appointment [22, 23], and the treatment category (e.g., surgery) [24], which was also consistent with our results. Regarding the relative importance of each variable in the model, the status of the last appointment, age, time of the day, lead time, and history of previous attendance are among the most important variables in the non-attendance predictive models presented in various analyses [12, 22, 25]. In our study, the history of previous attendance and the status of the last appointment also had a high weight in both models. In contrast, lead time and age were mainly important in pneumology and dermatology models, respectively. The time of the day had a small weight in both models.

Based on the performance results of the training algorithms, we chose decision trees to build our models, which was the second most frequently used algorithm to develop predictive models in the review of Carreras et al., after logistic regression [14]. The accuracy values reported in the review for models based on decision trees ranged from 76.5% to 89.6%, higher than the accuracy found in our analysis. However, most studies had a limited sample size and/or used the same dataset for training algorithms and assessing their performance. Alternatively to this approach, which may lead to overfitting, we used an independent dataset for model validation. Therefore, although lower than reported elsewhere, we think our results may better reflect the expected accuracy of the model when applied to the real-world.

Regardless of the validation approach, most studies reported accuracy values lower than the attendance rate [14]. This trend, also observed in our analysis, may be explained by the lack of data from other domains such as social, cultural, and socioeconomic factors that might have a relevant contribution to non-attendance behavior. Finally, we observed a poorer performance of the pneumology model compared with the dermatology model, which might also be due to differences in outpatient procedures and patient complexity between services. These findings suggest that service-specific characteristics and predictors from other domains should be included in the development of prediction models for non-attendance.

Like in our pilot study, other authors have reported non-attendance reductions after implementing reminding strategies based on phone calls [26] or, most frequently, short message services (SMS) [9–11]. However, phone calls are more expensive than SMS [9, 27], and both interventions have high costs for healthcare centers. Irrespective of the type of reminder, predictive algorithms may help to prioritize patients at higher risk of non-attendance, which is likely to improve the cost-effectiveness of the intervention. Furthermore, the quantitative approach to the prediction of non-attendance allows combining more or less compelling interventions based on different thresholds of non-attendance risk (e.g., SMS at risk between 50%-90%, and phone calls at risk ≥90%).

A remarkable consequence of our intervention for reducing non-attendance was the overloading of hospital agendas, highlighted during the debriefing held after the pilot study. This perception, which is consistent with the effectiveness of the measure, indicates that medical appointments were routinely scheduled on an overbooking basis, assuming certain level of non-attendance. Hence, the potential consequences of improving efficiency in healthcare systems should be considered before implementing these types of solutions. Another concern raised during the debriefing session was the cost (in terms of time spent by administrative staff) associated with phone calls to individuals at higher risk of non-attendance. The economic impact of this solution can be minimized by implementing call centers shared by various centers or investigating the optimal cut-off of non-attendance risk for a patient to be included in the intervention. For cut-off selection, other approaches like the efficiency curve (similar to the Lorenz curve used in economics) could be explored [28]. Nevertheless, cost-effectiveness analyses that consider the cost associated with non-attendance should be conducted before drawing conclusions on the actual economic impact of this intervention.

The interpretation of our results is limited by the simultaneous assessment of the predictive model and the intervention itself (i.e., phone call reminder), which precluded appraising the contribution of each feature to the non-attendance reduction. However, the main purpose of our pilot study was to assess the applicability of the whole concept to day-to-day practice. Another limitation was the unavailability of data with potential influence on the non-attendance rate, such as the economic status [29, 30], education level [31, 32], or certain medical conditions [20, 33]. As discussed previously, the lack of social information is common in the development of predictive algorithms elsewhere. Regardless of the future inclusion of these data, the model should undergo continual learning by retraining to assure its validity through time, including the seasonal perspective, which is likely to influence the outcomes. The model has to be aware of new patients or categorical features, as well as considering up-to-date data to include the latest trends of non-attendance in each hospital service. Alternative analytical approaches, such as logistic regression analysis, could also be explored.

## Conclusions

The results of our study show that the use of non-attendance predictive models can be a valuable tool to identify patients at higher risk of non-attending a medical appointment and should be, therefore, prioritized for active reminders such as phone calls. The overloading of the hospital agenda experienced as a consequence of the effectiveness of the intervention underscores the need to consider organizational changes when implementing interventions for reducing non attendance rates. The free availability of our algorithm warrants future research to adapt it to other patient profiles and assess the cost-effectiveness of interventions based patient stratification according to the risk of non-attendance.

## Supplementary Information

**Additional file 1.** Supplementary file 1.

## Data Availability

Supplementary Tables and methods are available in Supplementary file 1. The algorithms developed and presented in this manuscript are freely accessible from the following Github repository: https://github.com/gencat/outpatient-nonattendance-prediction. The local data protection framework does not allow public availability of the patient information used in this work.

## Abbreviations

ANOVA: Analysis of variance
CART: Classification and Regression Trees
CI: Confidence interval
kNN: k-nearest neighbor
RCT: Randomized controlled trial
SD: Standard deviation
SMS: Short message service
SVM: Support vector machines

## References

[1] LaGanga LR (2007). Clinic Overbooking to Improve Patient Access and Increase Provider Productivity*. Decis Sci..

[2] Bech M (2005). The economics of non-attendance and the expected effect of charging a fine on non-attendees. Health Policy (New York)..

[3] Dantas LF (2018). No-shows in appointment scheduling – a systematic literature review. Health Policy (New York)..

[4] Samorani M (2015). Outpatient appointment scheduling given individual day-dependent no-show predictions. Eur J Oper Res..

[5] Kopach R (2007). Effects of clinical characteristics on successful open access scheduling. Health Care Manag Sci..

[6] Hardy KJ (2001). Quality improvement report: Information given to patients before appointments and its effect on non-attendance rate. BMJ..

[7] Guy R (2012). How effective are short message service reminders at increasing clinic attendance? A meta-analysis and systematic review. Health Serv Res..

[8] Pollastri AR (2005). Incentive program decreases no-shows in nontreatment substance abuse research. Exp Clin Psychopharmacol..

[9] Chen Z (2008). Comparison of an SMS text messaging and phone reminder to improve attendance at a health promotion center: A randomized controlled trial. J Zhejiang Univ Sci B..

[10] Gurol-Urganci I. Mobile phone messaging reminders for attendance at healthcare appointments. Cochrane Database Syst Rev. 2013;2017(12) 10.1002/14651858.CD007458.pub3 PMC6485985 24310741

[11] Parikh A (2010). The Effectiveness of Outpatient Appointment Reminder Systems in Reducing No-Show Rates. Am J Med..

[12] Norris JB (2014). An empirical investigation into factors affecting patient cancellations and no-shows at outpatient clinics. Decis Support Syst..

[13] Torres O (2015). Risk factor model to predict a missed clinic appointment in an urban, academic, and underserved setting. Popul Health Manag..

[14] Carreras-García D. Patient no-show prediction: A systematic literature review. Entropy. 2020;22(6) 10.3390/e22060675 PMC7517206 33286447

[15] McMahan HB (2013). Ad click prediction: a view from the trenches. Proceedings of the 19th ACM SIGKDD international conference on Knowledge discovery and data mining.

[16] Awoyemi JO, Adetunmbi AO, Oluwadare SA. "Credit card fraud detection using machine learning techniques: A comparative analysis," 2017. International Conference on Computing Networking and Informatics (ICCNI). 2017:1-9. 10.1109/ICCNI.2017.8123782.

[17] Rudolph JW (2006). There’s no such thing as “nonjudgmental” debriefing: a theory and method for debriefing with good judgment. Simul Healthc..

[18] Ahmad MU. A predictive model for decreasing clinical no-show rates in a primary care setting. Int. J Healthc Manag. 2019;

[19] Gromisch ES. Who is not coming to clinic? A predictive model of excessive missed appointments in persons with multiple sclerosis. Mult Scler Relat Disord. 2020;38 10.1016/j.msard.2019.101513 31756611

[20] Daggy J (2010). Using no-show modeling to improve clinic performance. Health Inform J..

[21] Ding X (2018). Designing risk prediction models for ambulatory no-shows across different specialties and clinics. J Am Med Informatics Assoc..

[22] Elvira C (2018). Machine-Learning-Based No Show Prediction in Outpatient Visits. Int J Interact Multimed Artif Intell..

[23] Chua SL (2019). Development of predictive scoring model for risk stratification of no-show at a public hospital specialist outpatient clinic. Proc Singapore Healthc..

[24] Suk M-Y (2021). Evaluation of Patient No-Shows in a Tertiary Hospital: Focusing on Modes of Appointment-Making and Type of Appointment. Int J Environ Res Public Health..

[25] Chong LR. Artificial Intelligence Predictive Analytics in the Management of Outpatient MRI Appointment No-Shows. Am J Roentgenol. 2020:1–8. 10.2214/AJR.19.22594 32901567

[26] Lagman RL. “If You Call Them, They Will Come”: A Telephone Call Reminder to Decrease the No-Show Rate in an Outpatient Palliative Medicine Clinic. Am J Hosp Palliat Med. 2020; 10.1177/1049909120952322 32845702

[27] Leong KC (2006). The use of text messaging to improve attendance in primary care: A randomized controlled trial. Fam Pract..

[28] Prytherch DR (2010). ViEWS—towards a national early warning score for detecting adult inpatient deterioration. Resuscitation..

[29] Gomes MAG (2019). No-shows at public secondary dental care for pediatric patients: a cross-sectional study in a large Brazilian city. Cien Saude Colet..

[30] Miller AJ (2015). Predictors of repeated “no-showing” to clinic appointments. Am J Otolaryngol - Head Neck Med Surg..

[31] Jensen H. Characteristics of customary non-attenders in general practice who are diagnosed with cancer: A cross-sectional study in Denmark. Eur J Cancer Care (Engl). 2019;28(6) 10.1111/ecc.13143 31433525

[32] Wolff DL (2019). Rate and predictors for non-attendance of patients undergoing hospital outpatient treatment for chronic diseases: A register-based cohort study. BMC Health Serv Res..

[33] Coleman MM (2014). Injury type and emergency department management of orthopaedic patients influences follow-up rates. J Bone Jt Surg Am.

