# Supplementary material for: Reducing non-attendance in outpatient appointments: predictive model development, validation, and clinical assessment
Source: BMC Health Serv Res. 2022 Apr 6;22:451. doi: 10.1186/s12913-022-07865-y (PMC8985245; doi:10.1186/s12913-022-07865-y)
Supplement: Supplementary file 1 — Additional file 1. Supplementary file 1. [file 12913_2022_7865_MOESM1_ESM.docx]

**Preventing non-attendance in outpatient appointments: predictive model development, validation, and clinical assessment**

Supplementary file 1

Contents

[Supplementary Methods 2](#_Toc85694921)

[Selection of the training algorithm 2](#_Toc85694922)

[Supplementary Tables 3](#_Toc85694923)

[References 12](#_Toc85694924)

# Supplementary Methods

## Selection of the training algorithm

Table S1 shows the specificity, sensitivity, and accuracy of the different training algorithms. The algorithm kNN yielded unacceptable results in terms of sensitivity, whereas XGBoost and SVM resulted in similar metric performance values to those of decision trees. Based on the performance of each training algorithm, and also considering the easiness of explanation, the decision trees algorithm was selected.

Decision trees were trained using recursive partitioning within the R library rpart [1]. This library presents an implementation of Classification and Regression Trees (CART), described by Breiman L. et al [2]. The goodness of fit of each split was measured using the Gini impurity. The importance of each variable was calculated as the sum of the goodness of split measures for each split for which it was the primary variable, plus adjusted goodness agreement for all splits in which it was a surrogate. According to the 5-fold cross-validation, the values that optimized the trade-off between specificity, accuracy, and sensitivity of the models were a complexity factor of 0.01 and a maximum depth of the decision tree algorithm of 10.

A loss matrix was used to grow the trees, penalizing type II errors (i.e., false negatives) with a relative weight of 1.2. The relative weight of type I error was set to 1.

Table S1. Performance of different training algorithms (%)

|  | **Decision Trees** | **XGBoost** | **SVM** | **kNN** |
| --- | --- | --- | --- | --- |
| **Dermatology** |  |  |  |  |
| Specificity | 73.01 | 74.37 | 64.86 | 81.17 |
| Accuracy | 64.78 | 63.80 | 62.62 | 54.91 |
| Sensitivity | 56.56 | 53.22 | 60.63 | 19.38 |
|  |  |  |  |  |
| **Pneumology** |  |  |  |  |
| Specificity | 58.56 | 54.43 | 55.61 | 94.44 |
| Accuracy | 59.70 | 58.23 | 59.90 | 51.91 |
| Sensitivity | 60.85 | 62.03 | 64.20 | 9.38 |
|  |  |  |  |  |

kNN: k-nearest neighbor. SVM: support vector machines.

# Supplementary Tables

**Table S2.** Associations between variables and non-attendance for dermatology service (N=33329).

|  | **Attended appointments (N=26363)** | **Non-attended appointments (N=6966)** | ***P*** |
| --- | --- | --- | --- |
| **Categorical variables, *No (%)*** | | | |
| Gender (male) | 11943 (45.30) | 3148 (45.19) | 0.365 |
| Age range (years) | |  |  |
| 0-14 | 751 (2.85) | 290 (4.16) | **< 0.001** |
| 14-18 | 668 (2.53) | 193 (2.77) |  |
| 18-25 | 1998 (7.58) | 603 (8.66) |  |
| 25-35 | 1833 (6.95) | 911 (13.08) |  |
| 35-50 | 4147 (15.73) | 1542 (22.14) |  |
| 50-65 | 5366 (20.35) | 1303 (18.71) |  |
| 65-80 | 7084 (26.87) | 1227 (17.61) |  |
| > 80 | 4516 (17.13) | 897 (12.88) |  |
| Nationality | |  |  |
| Spanish | 18171 (68.93) | 4911 (70.5) | **< 0.001** |
| Not informed | 7846 (29.76) | 1886 (27.07) |  |
| Other | 346 (1.31) | 169 (2.43) |  |
| Marital status | |  |  |
| Single | 791 (3.00) | 263 (3.78) | 0.052 |
| Married | 1575 (5.97) | 301 (4.32) |  |
| Widowed | 16 (0.06) | 5 (0.07) |  |
| Divorced | 5 (0.02) | 3 (0.04) |  |
| Don't know/No opinion | 20957 (79.49) | 5217 (74.89) |  |
| Not available | 3019 (11.45) | 1177 (16.9) |  |
| Distance from home to hospital (Km) | | | |
| <1,2 | 3974 (15.07) | 1148 (16.48) | **0.009** |
| 1.2-3.5 | 127 (0.48) | 20 (0.29) |  |
| 3.5-20 | 3538 (13.42) | 886 (12.72) |  |
| 20-500 | 102 (0.39) | 23 (0.33) |  |
| >500 | 17991 (68.24) | 4644 (66.67) |  |
| Not available | 631 (2.39) | 245 (3.52) |  |
| Time range of the appointment (h) | | | |
| 08-10 am | 8975 (34.04) | 2176 (31.24) | **< 0.001** |
| 10-12 am | 9618 (36.48) | 2714 (38.96) |  |
| 12-2 pm | 6407 (24.30) | 1678 (24.09) |  |
| 2-4 pm | 877 (3.33) | 265 (3.80) |  |
| 4-6 pm | 485 (1.84) | 133 (1.91) |  |
| Day of the appointment | | |  |
| Monday | 4961 (18.82) | 1530 (21.96) | **< 0.001** |
| Tuesday | 201 (0.76) | 59 (0.85) |  |
| Wednesday | 8499 (32.24) | 2137 (30.68) |  |
| Thursday | 6608 (25.07) | 1549 (22.24) |  |
| Friday | 6094 (23.12) | 1689 (24.25) |  |
| Month of appointment | | |  |
| January | 2139 (8.11) | 642 (9.22) | **0.035** |
| February | 2668 (10.12) | 780 (11.20) |  |
| March | 2725 (10.34) | 662 (9.50) |  |
| April | 2553 (9.68) | 594 (8.53) |  |
| May | 2622 (9.95) | 608 (8.73) |  |
| June | 2553 (9.68) | 651 (9.35) |  |
| July | 2571 (9.75) | 641 (9.20) |  |
| August | 476 (1.81) | 115 (1.65) |  |
| September | 1870 (7.09) | 491 (7.05) |  |
| October | 2124 (8.06) | 667 (9.58) |  |
| November | 2761 (10.47) | 774 (11.11) |  |
| December | 1301 (4.93) | 341 (4.90) |  |
| Type of appointment | |  |  |
| First | 12929 (49.04) | 3676 (52.77) | **< 0.001** |
| Second | 3558 (13.50) | 1150 (16.51) |  |
| Successive | 9876 (37.46) | 2140 (30.72) |  |
| Reason of the appointment | | |  |
| Control | 11790 (44.72) | 2754 (39.53) | **< 0.001** |
| Quick diagnosis | 3 (0.01) | 1 (0.01) |  |
| Regular | 13826 (52.44) | 4034 (57.91) |  |
| Preferential | 719 (2.73) | 168 (2.41) |  |
| Not available | 25 (0.09) | 9 (0.13) |  |
| Treatment category | |  |  |
| First visit | 17937 (68.04) | 5689 (81.67) | **< 0.001** |
| First visit (hospital referral) | 12 (0.05) | 5 (0.07) |  |
| Second visit | 2088 (7.92) | 432 (6.2) |  |
| Second visit (special) | 2 (0.01) | 1 (0.01) |  |
| Ambulatory surgery | 5778 (21.92) | 653 (9.37) |  |
| Ambulatory care | 2 (0.01) | 0 (0.00) |  |
| Consultation | 340 (1.29) | 110 (1.58) |  |
| Other* | 204 (0.77) | 76 (1.09) |  |
| Physician** | |  |  |
| D-1 | 7409 (28.10) | 2037 (29.24) | **<0.001** |
| D-2 | 7308 (27.72) | 1849 (26.54) |  |
| D-3 | 11646 (44.18) | 3080 (44.21) |  |
| Attended last visit | 21612 (81.98) | 3572 (51.28) | **<0.001** |
| **Numerical variables, mean *(SD)*** | | | |
| Lead time (days)*** | 116.33 (107.98) | 132.72 (111.88) | **< 0.001** |
| Nº of reschedules | 0.01 (0.12) | 0.01 (0.11) | **0.020** |
| Rate of attendance of previous appointments | 0.85 (0.11) | 0.71 (0.22) | **< 0.001** |
| Nº of previous visits | 51.26 (50.6) | 38.51 (44.11) | **< 0.001** |
| Time from last visit (days) | 229.96 (264.78) | 336.65 (348.09) | **< 0.001** |
| *categories with a percentage < 1% in both services ** Arbitrary codes for dermatology (D) physicians ***days of waiting since scheduling until the appointment date | | | |

**Table S3.** Associations between variables and non-attendance for pneumology service (N=21050).

|  | **Attended appointments** (N=17184) | **Non-attended appointments** (N=3866) | ***P*** |
| --- | --- | --- | --- |
| **Categorical variables, *No (%)*** |  |  |  |
| Gender (male) | 10521 (61.23) | 2336 (60.42) | 0.885 |
| Age range (years) |  |  |  |
| 0-14 | 1 (0.01) | 0 (0.00) | **< 0.001** |
| 14-18 | 16 (0.09) | 2 (0.05) |  |
| 18-25 | 135 (0.79) | 40 (1.03) |  |
| 25-35 | 325 (1.89) | 169 (4.37) |  |
| 35-50 | 1470 (8.55) | 511 (13.22) |  |
| 50-65 | 4711 (27.42) | 1247 (32.26) |  |
| 65-80 | 7306 (42.52) | 1320 (34.14) |  |
| > 80 | 3220 (18.74) | 577 (14.92) |  |
| Nationality |  |  |  |
| Spanish | 13836 (80.52) | 3071 (79.44) | **< 0.001** |
| Not informed | 3119 (18.15) | 689 (17.82) |  |
| Other | 229 (1.33) | 106 (2.74) |  |
| Marital status |  |  |  |
| Single | 345 (2.01) | 86 (2.22) | **<0.001** |
| Married | 1494 (8.69) | 284 (7.35) |  |
| Widowed | 26 (0.15) | 7 (0.18) |  |
| Divorced | 17 (0.10) | 0 (0.00) |  |
| Don't know/No opinion | 14127 (82.21) | 3073 (79.49) |  |
| Not available | 1175 (6.84) | 416 (10.76) |  |
| Distance from home to hospital (Km) |  |  |  |
| <1,2 | 2538 (14.77) | 641 (16.58) | **0.003** |
| 1.2-3.5 | 128 (0.74) | 29 (0.75) |  |
| 3.5-20 | 2117 (12.32) | 419 (10.84) |  |
| 20-500 | 68 (0.4) | 16 (0.41) |  |
| >500 | 12243 (71.25) | 2711 (70.12) |  |
| Not available | 90 (0.52) | 50 (1.29) |  |
| Time range of the appointment (h) |  |  |  |
| 08-10 am | 3501 (20.37) | 688 (17.80) | **< 0.001** |
| 10-12 am | 6631 (38.59) | 1409 (36.45) |  |
| 12-2 pm | 4537 (26.40) | 921 (23.82) |  |
| 2-4 pm | 1538 (8.95) | 525 (13.58) |  |
| 4-6 pm | 977 (5.69) | 323 (8.35) |  |
| Day of the appointment |  |  |  |
| Monday | 3583 (20.85) | 932 (24.11) | **< 0.001** |
| Tuesday | 3793 (22.07) | 947 (24.5) |  |
| Wednesday | 4062 (23.64) | 752 (19.45) |  |
| Thursday | 2564 (14.92) | 651 (16.84) |  |
| Friday | 3182 (18.52) | 584 (15.11) |  |
| Month of appointment |  |  |  |
| January | 1548 (9.01) | 344 (8.90) | **<0.001** |
| February | 1662 (9.67) | 398 (10.29) |  |
| March | 1554 (9.04) | 349 (9.03) |  |
| April | 1529 (8.90) | 357 (9.23) |  |
| May | 1722 (10.02) | 383 (9.91) |  |
| June | 1662 (9.67) | 351 (9.08) |  |
| July | 1471 (8.56) | 270 (6.98) |  |
| August | 405 (2.36) | 118 (3.05) |  |
| September | 1344 (7.82) | 319 (8.25) |  |
| October | 1730 (10.07) | 388 (10.04) |  |
| November | 1695 (9.86) | 376 (9.73) |  |
| December | 862 (5.02) | 213 (5.51) |  |
| Type of appointment |  |  |  |
| First | 2892 (16.83) | 889 (23.00) | **< 0.001** |
| Second | 1915 (11.14) | 407 (10.53) |  |
| Successive | 12377 (72.03) | 2570 (66.48) |  |
| Reason of the appointment |  |  |  |
| Control | 10458 (60.86) | 2070 (53.54) | **< 0.001** |
| Quick diagnosis | 244 (1.42) | 35 (0.91) |  |
| Regular | 2975 (17.31) | 932 (24.11) |  |
| Preferential | 321 (1.87) | 49 (1.27) |  |
| Not available | 3186 (18.54) | 780 (20.18) |  |
| Treatment category |  |  |  |
| First visit | 10077 (58.64) | 2376 (61.46) | **< 0.001** |
| First visit (hospital referral) | 527 (3.07) | 79 (2.04) |  |
| Second visit | 1587 (9.24) | 319 (8.25) |  |
| Second visit (special) | 2542 (14.79) | 571 (14.77) |  |
| Ambulatory surgery | 29 (0.17) | 2 (0.05) |  |
| Ambulatory care | 384 (2.23) | 61 (1.58) |  |
| Consultation | 1203 (7.00) | 258 (6.67) |  |
| Polysomnography | 168 (0.98) | 48 (1.24) |  |
| Other* | 667 (3.88) | 152 (3.93) |  |
| Physician** |  |  |  |
| P-1 | 4355 (25.34) | 1118 (28.92) | 0.070 |
| P-2 | 2189 (12.74) | 394 (10.19) |  |
| P-3 | 3221 (18.74) | 806 (20.85) |  |
| P-4 | 341 (1.98) | 78 (2.02) |  |
| P-5 | 4813 (28.01) | 1096 (28.35) |  |
| P-6 | 1142 (6.65) | 146 (3.78) |  |
| Not available | 1123 (6.54) | 228 (5.90) |  |
| Attended last visit | 13591 (79.09) | 2284 (59.08) | **<0.001** |
| **Numerical variables, mean *(SD)*** |  |  |  |
| Lead time (days)*** | 114.73 (129.81) | 145.53 (139.54) | **< 0.001** |
| Nº of reschedules | 0.03 (0.16) | 0.03 (0.17) | 0.340 |
| Rate of attendance of previous appointments | 0.84 (0.09) | 0.76 (0.16) | **< 0.001** |
| Nº of previous visits | 96.18 (96.32) | 77.62 (81.09) | **< 0.001** |
| Time from last visit (days) | 155.9 (216.74) | 241.33 (314) | **< 0.001** |
| *categories with a percentage < 1% in both services ** Arbitrary codes for pneumology (P) physicians ***days of waiting since scheduling until the appointment date | | | |

**Table S4.** Correlation matrix among variables influencing non-attendance

| **A) Dermatology** | | | | | | | | | | | |
| --- | --- | --- | --- | --- | --- | --- | --- | --- | --- | --- | --- |
| **Categorical variables** | Age range | Nationality | Marital status | Distance home-hospital | Time of appointment | Day of appointment | Month of appointment | Type of visit | Reason of appointment | Treatment category | Physician |
| Nationality | 0.13 |  |  |  |  |  |  |  |  |  |  |
| Marital status | 0.15 | 0.04 |  |  |  |  |  |  |  |  |  |
| Distance home-hospital | 0.04 | 0.05 | 0.03 |  |  |  |  |  |  |  |  |
| Time of appointment | 0.06 | 0.02 | 0.02 | 0.04 |  |  |  |  |  |  |  |
| Day of appointment | 0.03 | 0.01 | 0.02 | 0.13 | 0.25 |  |  |  |  |  |  |
| Month of appointment | 0.02 | 0.02 | 0.02 | 0.03 | 0.03 | 0.07 |  |  |  |  |  |
| Type of appointment | 0.12 | 0.02 | 0.02 | 0.04 | 0.16 | 0.06 | 0.03 |  |  |  |  |
| Reason of appointment | 0.10 | 0.02 | 0.02 | 0.03 | 0.16 | 0.05 | 0.04 | 0.59 |  |  |  |
| Treatment category | 0.08 | 0.06 | 0.04 | 0.07 | 0.06 | 0.05 | 0.03 | 0.16 | 0.17 |  |  |
| Physician | 0.05 | 0.02 | 0.03 | 0.30 | 0.19 | 0.28 | 0.13 | 0.04 | 0.03 | 0.15 |  |
| Attendance of last appointment | 0.17 | 0.05 | 0.05 | 0.04 | 0.02 | 0.02 | 0.02 | 0.04 | 0.03 | 0.07 | 0.02 |
| **Numerical variables** | Lead time | Nº reschedules | Rate of previous attendance | Nº previous appointments |  |  |  |  |  |  |  |
| Nº reschedules | 0.03 |  |  |  |  |  |  |  |  |  |  |
| Attendance of previous appointments | 0.04 | 0.01 |  |  |  |  |  |  |  |  |  |
| Nº previous appointments | 0.09 | 0.01 | 0.04 |  |  |  |  |  |  |  |  |
| Time from last appointment | 0.00 | -0.06 | -0.04 | -0.53 |  |  |  |  |  |  |  |

| **B) Pneumology** | | | | | | | | | | | |
| --- | --- | --- | --- | --- | --- | --- | --- | --- | --- | --- | --- |
| **Categorical variables** | Age range | Nationality | Marital status | Distance home-hospital | Time of appointment | Day of appointment | Month of appointment | Type of visit | Reason of appointment | Treatment category | Physician |
| Nationality | 0.16 |  |  |  |  |  |  |  |  |  |  |
| Marital status | 0.13 | 0.07 |  |  |  |  |  |  |  |  |  |
| Distance home-hospital | 0.06 | 0.06 | 0.04 |  |  |  |  |  |  |  |  |
| Time of appointment | 0.07 | 0.05 | 0.03 | 0.03 |  |  |  |  |  |  |  |
| Day of appointment | 0.10 | 0.05 | 0.03 | 0.03 | 0.19 |  |  |  |  |  |  |
| Month of appointment | 0.03 | 0.03 | 0.03 | 0.02 | 0.05 | 0.03 |  |  |  |  |  |
| Type of appointment | 0.12 | 0.05 | 0.04 | 0.04 | 0.18 | 0.11 | 0.05 |  |  |  |  |
| Reason of appointment | 0.09 | 0.05 | 0.03 | 0.04 | 0.22 | 0.12 | 0.05 | 0.56 |  |  |  |
| Treatment category | 0.12 | 0.12 | 0.07 | 0.15 | 0.12 | 0.12 | 0.05 | 0.17 | 0.17 |  |  |
| Physician | 0.09 | 0.07 | 0.04 | 0.08 | 0.28 | 0.20 | 0.08 | 0.30 | 0.34 | 0.21 |  |
| Attendance last appointment | 0.11 | 0.04 | 0.04 | 0.09 | 0.04 | 0.04 | 0.05 | 0.06 | 0.06 | 0.13 | 0.06 |
| **Numerical variables** | Lead time | Nº reschedules | Rate of previous attendance | Nº previous appointments |  |  |  |  |  |  |  |
| Rate of previous attendance | 0.03 |  |  |  |  |  |  |  |  |  |  |
| Nº previous appointments | -0.12 | 0.07 |  |  |  |  |  |  |  |  |  |
| Time from last appointment | 0.11 | -0.07 | -0.46 |  |  |  |  |  |  |  |  |

# References

1. Therneau T. rpart: Recursive partitioning for classification, regression and survival trees. CRAN R package version 4.1-15. 2019.

2. Breiman L. Classification and Regression Trees (Wadsworth Statistics/Probability). New York CRC Press. 1984;
